# Early Pandemic Improvements in Diet Quality Are Associated with Increased Physical Activity and Weight Loss in US Adults

**DOI:** 10.3390/ijerph19148289

**Published:** 2022-07-07

**Authors:** Corinne E. Gautreaux, Kristen S. Smith, Luke Dolan, Michael B. Marlin, Michael W. Greene, Josh R. Novak, Andrew D. Frugé

**Affiliations:** 1 College of Science and Mathematics, Auburn University, 315 Roosevelt Concourse, Auburn, AL 36849, USA; ceg0054@auburn.edu; 2 College of Human Sciences, Auburn University, 260 Mell Street, Auburn, AL 36849, USA; kss0034@auburn.edu (K.S.S.); mwg0006@auburn.edu (M.W.G.); jrn0031@auburn.edu (J.R.N.); 3 Office of the Dean of Students, University of Alabama, 751 Campus Drive, Tuscaloosa, AL 35487, USA; ladolan@ua.edu; 4 Department of Emergency Medicine, University of Mississippi Medical Center, 2500 North State Street, Jackson, MS 39216, USA; mmarlin@umc.edu; 5 College of Nursing, Auburn University, 710 South Donahue Drive, Auburn, AL 36849, USA

**Keywords:** diet, physical activity, pandemic, COVID-19, body mass index

## Abstract

In March 2020, the COVID-19 pandemic led to restricted vocational (Voc-PA) and recreational physical activity (Rec-PA) outside of the home. We conducted a nation-wide survey in the United States (US) during the mitigation peak of the pandemic (June 2020) to assess health-related changes from the previous year. A diet quality (DQ) assessment tool weighted the relative healthfulness of eating occasions from foods prepared-at-home (Home) and away-from-home (Away). Previously-validated instruments assessed PA and demographic variables; height/weight were self-reported to calculate body mass index (BMI). T-tests explored longitudinal, between-sex, and obesity status differences in DQ, PA, and BMI; Pearson correlations explored associations. Of 1648 respondents, 814 valid responses (56.8% female, 81.7% white) were analyzed. Overall and Home DQ was higher for females than males in 2020 (*p* < 0.001 for both). Respondents increased DQ from 2019 to 2020, primarily from Away (*p* < 0.001 for both sexes). Total Rec-PA and Voc-PA was higher in males (*p* = 0.002, *p* < 0.001) than females in 2020; females reported higher other PA (*p* = 0.001). Change in BMI was inversely associated with change in both DQ and PA (*p* < 0.001 for both). In this sample of US adults, early adaptations to the COVID-19 pandemic included improved DQ and BMI. Whether these short-term improvements were maintained warrant further investigation.

## 1. Introduction

Severe acute respiratory syndrome coronavirus 2 (SARS-CoV-2) is the highly infectious virus responsible for the current pandemic of COVID-19. This infection spreads rapidly and is highly transmissible due to person-to-person spread, including among asymptomatic persons [1]. The first cases of COVID-19 were observed in Wuhan, China in December of 2019, and COVID-19 was classified as a global pandemic in the early months of 2020. In March 2020, COVID-19 spread across the United States, overwhelming the most densely populated cities, which led to subsequent changes in vocational and recreational activities due to a disruption in daily life [2]. Several factors, including mandatory quarantine periods, restriction of travel, closing of businesses, curfews, and restricted outdoor activities, resulted in increased time spent at home and indoors [3]. The potential negative effects of quarantine due to the pandemic include impacts on diet quality (DQ), physical activity (PA), and weight change.

An increase in the quantity of food intake through snacking and an increase in number of meals eaten due to the psychological impacts of confinement such as loneliness, stress, boredom, and anxiety has been observed in international populations [4]. According to an international survey aiming to assess lifestyle, eating habit changes, and psychological and emotional aspects caused by social isolation, quality of diet worsened during the pandemic compared to before the pandemic [5], however this could be due to demographic and geographical factors. In fact, one notable observation was that greater control of over-eating was associated with lower age, lower body mass index (BMI; weight [kg]/height [m^2^]), and engagement in dieting prior to COVID-19 [5]. In Poland, a greater percentage of adults with BMI > 25 reported increased food consumption, snacking, and cooking compared to under- or normal weight individuals [6]. A common theme for populations of countries in Europe, Asia, and South America was increased eating frequency through meals and snacking [7]. One United States (US) study reported that dietary and eating habits have been impacted by eating more often with friends and family, eating in response to sight and smell of food, eating because you crave certain foods, eating in response to stress, eating when bored, and snacking more after dinner [8]. Thus, simply having greater access and/or fewer barriers to eating may promote caloric overconsumption.

Additionally, limited access to recreational facilities and normal vocational activities may have led to decreases in PA, although this seemed to be dependent on previous activity levels. In a Canadian population, it was shown that a high percentage of physically active individuals became more active, while those who were physically inactive became less active [9]. In a Spanish population, nearly half of respondents did not participate in physical activities during the confinement period [10]. In an international survey, home confinement as a result of the pandemic had a negative impact on physical activity at all intensity levels [11].

Heterogeneous effects of the pandemic on bodyweight have been reported. One study observed that individuals who were previously underweight (BMI < 18 kg/m^2^) or of normal weight (BMI 18–25 kg/m^2^) experienced a trend in weight loss whereas those who previously had BMI > 25 were more likely to gain weight [10]. In the US, it was observed that approximately 22% of US adults reported weight gain of 5–10 pounds throughout the COVID-19 pandemic, citing interrupted sleep schedules, increased snacking throughout the day, and stress eating [8].

We sought to examine DQ and PA of adult males and females during the mitigation peak in COVID-19 cases in the U.S. (June 2020) and describe these in the context of self-reported behaviors from the previous year. We further explored components of these behaviors relative to self-reported BMI change.

## 2. Materials and Methods

### 2.1. Subjects and Survey Distribution

Auburn University’s Institutional Review Board granted approval for this study. Participants were informed about confidentiality and their right to discontinue at any time prior to completing the survey. Participant consent was inferred by commencement of the survey. Recruitment of participants took place from 26–29 June 2020 through the online portal Amazon Mechanical Turk to complete the survey instrument [12,13]. Requirements for survey participation were to be 18 years of age or older along with a requirement to reside within the United States and have a HIT Approval Rate >98%. The survey was administered through Qualtrics, and participants were compensated 0.50 USD upon completion of a survey that passed initial quality control screening. Average time per assignment was 18 min, 14 s.

### 2.2. Survey Instrument

The survey instrument included several components which aimed to answer different research questions. Elements not included in this study were previously reported by Dolan et al. and discrepancies in inclusion/exclusion criteria resulted in different final sample sizes [12]. Fourteen questions were modified from the revised Morgenstern PA questionnaire (PAQ-M) by Rubenstein et al. in 2011 [14] to assess time spent in different activities during the previous week. Fifteen questions were included from the NCI Dietary Health Questionnaire (DHQ II) [15]. Fourteen questions were included from the U.S. Department of Commerce, U.S. Census Bureau’s 2020 Household Pulse Survey to collect demographic data, which guided reporting of race, ethnicity, and education categories. Height and weight were self-reported, and two questions were added and designed to assess diet quality by measuring the number of meals consumed per week by content and means of obtainment and/or preparation. Lastly, three questions served as attention check questions to screen for invalid responses [12]. Additional survey questions allowed respondents to self-assess their behaviors relative to the previous (2019) year.

### 2.3. Diet Quality Scoring

Diet quality questions assessed weekly number and type of meals eaten which were prepared-at-home (Home) or away-from-home (Away). Each type of meal was assigned a value based on relative healthfulness, as determined by a panel of three nutrition professionals, with the highest score awarded to the meal that was the most healthful (i.e., for meals prepared away from home: vegetarian/vegan-based entrée [7 score], pasta/noodle/rice-based mixed dish [6 score], seafood-based entrée [5 score], deli foods [soups, sandwiches, and salads] [4 score], meat or poultry-based entrée [3 score], pizza [2 score], and combination meals [burger or chicken sandwich and fries] [1 score]). The weekly percent of each meal type was then multiplied by the assigned code to obtain composite Home and Away DQ score. Composite DQ was determined with the equation Σ(% of meals per week per category × relative healthfulness score))/total # of eating occasions per week.

Perceived change in eating habits over the last year were assessed with the question “Compared to this time last year, would you say that your dietary habits have …” with 7 responses ranging from “severely declined” to “severely improved” which were used to determine face validity the change in Composite DQ.

### 2.4. Physical Activity Scoring

PA scores for 2020 were quantified by using the median value of the reported range of hours performed per week (i.e., 4 h, if survey response was 3–5 h per week) included in work (Voc-PA), exercise (Rec-PA), and other household activities to calculate weekly metabolic equivalents (METs) for each task. Each task was assigned a METs value from the previously validated physical activity questionnaire [14]. Change in PA was assessed by 7-item likert scale responses to the question: “Compared to this time last year, how would you say your exercise habits have changed?”.

### 2.5. Weight Change Scoring

BMI was calculated from self-reported height and weight (wt) for 2019 and 2020, and percent wt change determined using (2020 wt − 2019 wt)/2019 wt.

### 2.6. Quality Control

Valid survey responses were determined through several different measures. Participants were excluded based on the following criteria:

(1) Surveys with incomplete responses or incorrect/duplicate mTurk codes were excluded as were surveys with IP addresses outside of the United States.
(2) Three attention check questions were spread throughout the survey in the form of “answer x to this question”. Any incorrect answer to any of the three attention check questions led to response exclusion.
(3) Those individuals whose self-reported height/weight caused their BMI to be less than 14 or greater than 60 kg/m^2^ were excluded due to the implausibility of their BMI status.
(4) Individuals reporting physical activity hours that were more than the possible 24 h in a day were excluded from analyses.
(5) Participants were excluded if they reported fewer than 7 or greater than 70 eating occasions per week in the matrix response including sources and types of meals.

### 2.7. Statistical Analysis

Statistical analyses were conducted in SPSS version 28.0 (IBM Corp., Armonk, NY, USA). Descriptive statistics were obtained from demographic variables. Paired sample T-tests explored longitudinal differences between sexes in DQ, PA, and BMI and independent samples T-tests assessed between-sex and between-obesity category differences in DQ and PA. Pearson correlations assessed the relationships between body mass index, diet, and physical activity. Face validity of the DQ score was assessed with Pearson correlation between change in Composite DQ and self-reported change in DQ.

## 3. Results

**Figure 1:**
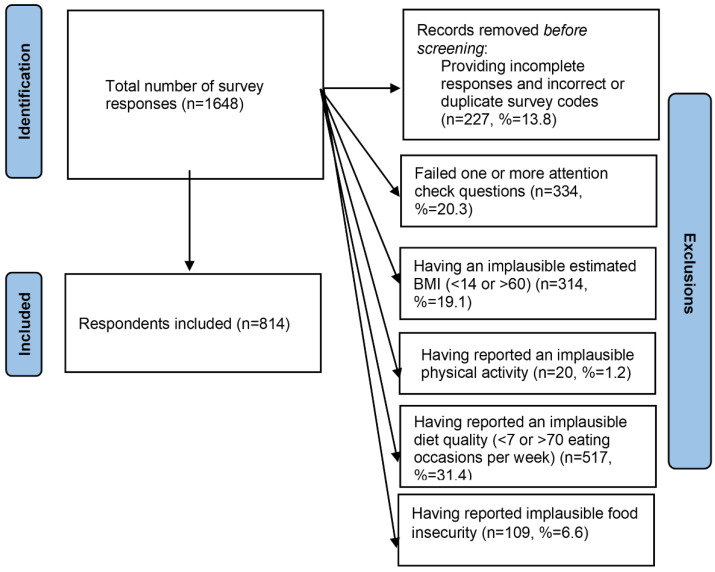
Flow of respondents and exclusions. Several responses met multiple exclusion criteria, thus the sum of exclusions exceeds 834.

A total of 1648 responses were collected, and due to the exclusion criteria outlined in Figure 1, the final sample size was 814.

The most common reason for exclusion was failing one or more attention check questions. The respondents included were 56.8% female, 38.0% between the ages of 18–34, 81.7% white, 40.2% completed a four-year degree, 47.4% currently married, and 85.6% currently employed (Table 1).

Changes in DQ, PA, and BMI between 2019 and 2020 and between-sex comparisons are reported in Table 2. Females showed more improvement than males in DQ scores both at home and overall DQ (*p* < 0.001 for both). Males, on the other hand, increased their DQ more than females in meals eaten Away (i.e., at a restaurant). All respondents significantly increased DQ from 2019 to 2020, with greatest improvements observed in Away meals (*p* < 0.001 for both sexes).

Improvements in PA from 2019 to 2020 were reported for 41.6% of females (*n* = 192) and 38.4% of males (*n* = 135). Rec-PA (*p* = 0.002) and Voc-PA (*p* < 0.001) were higher in males than females in 2020, though females reported higher PA from other modalities (*p* = 0.001). Hours sitting were equivalent for both sexes (*p* = 0.923).

**Figure 2:**
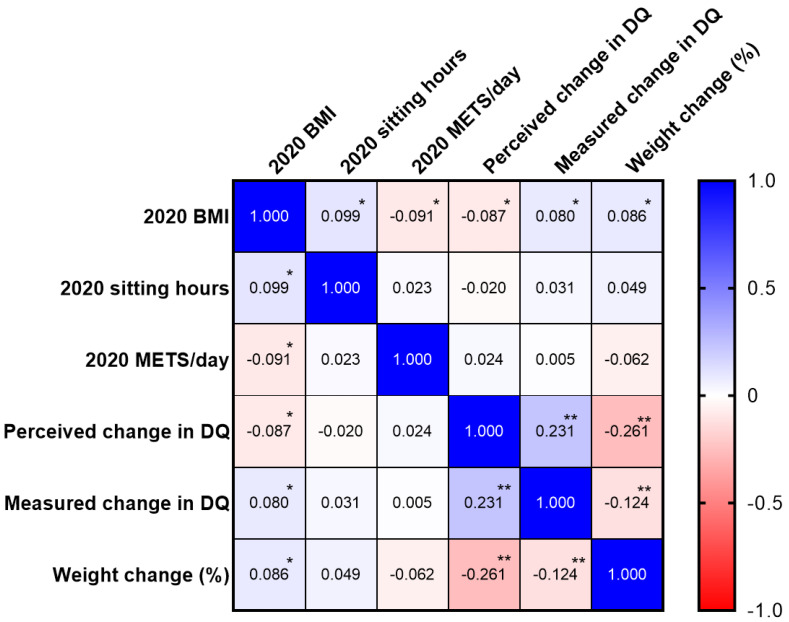
Heatmap of correlations between outcome variables. * *p*-values < 0.05. ** *p*-values < 0.001.

There were no differences in BMI (*p* = 0.078) between sexes. Significant reductions in BMI were observed in both sexes from the previous year (*p* = 0.019 for females; *p* = 0.01 for males). Correlations between outcome variables are reported in Figure 2. BMI in 2020 was positively associated with sitting hours (*p* = 0.005) and measured change in DQ (*p* = 0.022), and negatively associated with daily METs (*p* = 0.010) and perceived change in DQ (*p* = 0.013). Weight change was positively associated with BMI in 2020 (*p* = 0.014) and inversely associated with both perceived and measured change in DQ (*p* < 0.0001 for both). Finally, changes in measured DQ and perceived DQ were positively correlated (*p* < 0.0001), indicating our DQ Questionnaire was valid.

**Figure 3:**
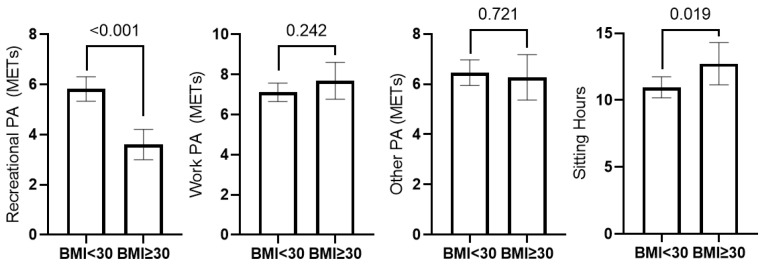
Physical Activity (PA) types in daily Metabolic Equivalents (METs) and daily sitting time in hours by obesity status.

Total Daily METs did not differ between individuals with and without obesity; however, those with BMI < 30 reported greater Rec-PA (*p* < 0.001) and less sitting time (*p* = 0.019) than those with obesity in 2020 (Figure 3). Additionally, participants with obesity reported greater weight loss (mean wt change: −2.64 kg ± 9.31 kg) than those with BMI < 30 (mean wt change: 0.1263 kg ± 6.42 kg) from 2019–2020 (*p* < 0.001).

## 4. Discussion

In our sample of US adults, survey participants generally improved their DQ from 2019–2020. Females reported higher at-home and overall DQ than males. Both sexes improved DQ from June 2019 to June 2020. These results were unexpected based on previous research that indicated DQ decreased in several global populations following COVID lockdowns. In studies conducted in both Spain and Italy, it was noted that greater than 50% of the surveyed population altered their eating habits throughout the lockdown period, with more negative than positive changes reported [10]. The decline in DQ seen in other populations could be attributed to increased snacking and increased number of meals eaten throughout the day due to the availability and accessibility of food resulting from more hours spent at home [4,5]. The global literature review by Zupo et al. reported overall increase in food intake including sugary foods, fruits, vegetables, cereals, junk/fast food, protein sources, and snack foods [7]. This review was conducted 10 August 2020 and cites papers with data collection primarily from March to May where fear and anxiety may have played a greater role in health behaviors. Conversely, the timing may also have been viewed as a welcome break from normal activities and been perceived as more of a vacation. While our results indicate an overall increase in diet quality consumed at home, our approach may have poorly captured snacking occasions. Additionally, differences in demographics and socioeconomic status of participants across the globe may have partially explain dissimilar observations.

An important factor to consider is the durability of behavior change and improvement. When the survey instrument was distributed, the US was experiencing the summer 2020 peak of the pandemic, and this included some of the harshest restrictions on the population. At this time, citizens were adjusting to a new normal and were closely adhering to the guidelines set forth by the World Health Organization and the Centers for Disease Control and Prevention. A few months later, an uptick in delivery meal services compensated for the strict restrictions, and therefore food was more accessible. This could have affected participants’ ability or willingness to maintain their improved DQ observed in this study. As restrictions continued to be altered whether that be becoming more strict or more lenient throughout the pandemic, PA levels could have fluctuated in the months following the conclusion of this study.

A large portion of our sample (38.4% of males and 41.6% of females) stated that their 2020 PA increased from 2019. One study by Lesser and Nienhuis showed participants who were previously physically active increased their PA during the pandemic, but those who were previously physically inactive showed a decline in PA due to pandemic restrictions [9]. Posthoc analyses from our data indicate 2020 Total METs were directly associated with perceived change in PA (r = 0.182, *p* < 0.001). While this does not support that observation directly, it would be plausible that individuals already exercising would use additional free time to devote more time to pursuing that hobby. Our participants with BMI < 30 reportedly engaged in more Rec-PA compared to participants with obesity, which support the observations by Lesser and Nienhuis et al. Interestingly, our participants with obesity reported greater weight loss over the past year, although they did not engage in more PA of any category than those with BMI < 30. It is likely that our limited assessment of their 2019 PA levels prohibited us from observing positive PA changes that would lead to their reported weight changes. Conversely, access to restaurants with foods containing greater caloric density may have been limited as well. Several of our findings differ from what has previously been reported, particularly with regard to PA. Ammar et al. reported a decrease in both time and intensity dedicated to PA in international populations [11]. It is possible that individuals anticipated that the COVID-19 restrictions would be short-term, and welcomed the break if exercising was not a hobby. It is unknown whether these PA changes observed in this study were maintained long-term as the pandemic continued to progress.

Herein, we observed reductions in BMI for both sexes from 2019–2020. However, while this was statistically significant, the weight reduction does not correspond to a clinically significant weight loss of 5% or more that would confer metabolic improvements and disease risk reduction [16]. Our observation that changes in BMI were inversely associated with DQ and positively associated with change in PA indicate our findings are reliable. One international study reported a higher proportion of individuals had gained rather than lost weight throughout the COVID-19 lockdown. Another study of a US population showed that nearly a quarter of US adults experienced a 5–10 pound weight gain throughout the COVID-19 pandemic [8]. It has been postulated that the increase in BMI shown in other populations both within the US and internationally could be linked to interruptions in other health behaviors such as interruptions in sleep schedules, increased food intake due to factors such as stress and emotions, and other psychological variables such as anxiety and depression [8]. The results shown in these other studies differ from the current study, as we observed that males and females in our sample experienced a slight decrease in weight during the pandemic.

Several limitations must be acknowledged. Our survey relied solely on self-reported data from participants, and implicit bias is inevitable with subjective data. Misreported information or false disclosures are always possible when distributing an online survey. Due to our stringent criteria for reliability, which omitted 50.6% of responses, we believe misreporting has been minimized. Second, the demographics of the included participants is not representative of the current US population, as the majority of the participants were white, between the ages of 18–34, completed a four-year degree and were currently employed. This is, instead, likely representative of MTurk users and our results are not generalizable [17]. Another limitation is the timeframe of our survey, which was distributed in June 2020. Subsequent peaks and surges lead to further lockdowns and social distancing guidelines, and behavior changes during these time periods were not measured. Therefore, we cannot confidently report that these positive behavior changes were sustained.

## 5. Conclusions

This survey of US adults indicates that the majority of this sample of individuals improved DQ and bodyweight as an early adaptation to the COVID-19 pandemic social distancing and isolation guidelines. Further investigation is necessary to determine whether these changes were maintained as the COVID-19 pandemic progressed. Because future pandemics are likely, public health messages focused on improving DQ and PA during restricted movement are warranted.

## Figures and Tables

**Table 1:** Characteristics of included study participants.

Characteristic	N	%
Sex		
Female	462	56.8
Male	352	43.2
Age		
18–34 years	309	38.0
35–44 years	279	34.3
45+ years	226	27.8
Race		
White	665	81.7
Black or African American	48	5.9
Asian	67	8.2
American Indian or Alaskan Native	8	1.0
Two or More Races	26	3.2
Hispanic, Latino, or Spanish Origin	51	6.3
Education		
High School or Less	313	38.5
Four-year degree	327	40.2
Greater than Four-year degree	174	21.4
Marital Status		
Not Married (Single, Widowed, Divorced, Separated)	428	52.6
Married	386	47.4
Employment Status		
Currently Employed	697	85.6
Currently Unemployed	117	14.4
Body Mass Index > 30 kg/m^2^ (2019)	207	25.4

**Table 2:** Diet quality, physical activity and body mass index of US men and women in June 2019 and June 2020.

	Females (*n* = 462)			Males (*n* = 352)			Sex Differences	
Diet Quality (DQ)	2020 Values	Change from 2019	*p*-Values	2020 Values	Change from 2019	*p*-Values	2020 Values	Change from 2019
Home DQ *	5.6 (1.0)	0.3 (0.9)	<0.001	5.2 (1.1)	0.1 (0.9)	0.090	<0.001	0.030
Away DQ *	5.1 (2.5)	0.9 (2.1)	<0.001	4.8 (2.5)	0.8 (2.2)	<0.001	0.222	0.381
Composite DQ *	5.4 (1.0)	0.4 (0.8)	<0.001	5 (1.1)	0.3 (0.8)	<0.001	<0.001	0.047
Physical Activity (PA)		Improving *n* (%)			Improving *n* (%)			
Average METs ^1,^*	37.1 (8.1)	192 (41.6)		38.3 (9.5)	135 (38.4)		0.054	
Recreational PA ^2,^*	33.1 (34.1)			42.3 (48.2)			0.002	
Vocational PA ^2,^*	46 (39.1)			57 (44.5)			<0.001	
Other PA ^2,^*	49.4 (49.3)			38.9 (38.3)			0.001	
Sitting Hours *	11.4 (10.3)			11.4 (10.5)			0.923	
Body Mass Index *	26.6 (7.0)	−0.5 (7.9)	0.019	27.4 (6.3)	−0.7 (6.6)	0.01	0.078	0.747

^1^ METs; Metabolic Equivalents; ^2^ METs per week. * Values reported as Mean (standard deviation) unless otherwise noted.

## Data Availability

The corresponding author can be contacted with data inquiries.
